# Impact of human immunodeficiency virus on pulmonary vascular disease

**DOI:** 10.21542/gcsp.2021.12

**Published:** 2021-06-30

**Authors:** Ashok Kumar, Aatish Mahajan, Ethan A. Salazar, Kevin Pruitt, Christian Arce Guzman, Matthias A. Clauss, Sharilyn Almodovar, Navneet K. Dhillon

**Affiliations:** 1 Pulmonary, Critical Care and Sleep Medicine, University of Kansas Medical Center, Kansas City, Kansas, USA; 2 Department of Immunology & Molecular Microbiology, Texas Tech University Health Sciences Center, Lubbock, Texas, USA; 3 Pulmonary, Critical Care, Sleep & Occupational Medicine, Indiana University School of Medicine, Indianapolis, Indiana, USA

## Abstract

With the advent of anti-retroviral therapy, non-AIDS-related comorbidities have increased in people living with HIV. Among these comorbidities, pulmonary hypertension (PH) is one of the most common causes of morbidity and mortality. Although chronic HIV-1 infection is independently associated with the development of pulmonary arterial hypertension, PH in people living with HIV may also be the outcome of various co-morbidities commonly observed in these individuals including chronic obstructive pulmonary disease, left heart disease and co-infections. In addition, the association of these co-morbidities and other risk factors, such as illicit drug use, can exacerbate the development of pulmonary vascular disease. This review will focus on these complex interactions contributing to PH development and exacerbation in HIV patients. We also examine the interactions of HIV proteins, including Nef, Tat, and gp120 in the pulmonary vasculature and how these proteins alter the endothelial and smooth muscle function by transforming them into susceptible PH phenotype. The review also discusses the available infectious and non-infectious animal models to study HIV-associated PAH, highlighting the advantages and disadvantages of each model, along with their ability to mimic the clinical manifestations of HIV-PAH.

## Introduction

HIV-infected patients with controlled viral load currently have a life span comparable to a normal person. Unfortunately, this increased life span is associated with chronic non-infectious co-morbidities that have introduced new challenges in the care and treatment of individuals living with HIV.

Among these co-morbidities, cardiovascular disease (CVD) and chronic pulmonary diseases such as pulmonary hypertension (PH), chronic obstructive pulmonary disease (COPD), gas exchange abnormalities, and asthma are most prevalent. Persistent chronic inflammatory conditions, dysregulated immune system, and oxidative stress leading to endothelial dysfunction, are some of the most studied factors responsible for HIV-associated chronic pulmonary complications. Among various associated pulmonary diseases, pulmonary arterial hypertension (PAH) is considered to be the most devastating, with the worst prognosis and survival rates.

Although chronic HIV-1 infection is independently associated with the development of Group I pulmonary hypertension (PH), the possibility of the presence of Group II and Group III forms of PH, due to left heart disease and chronic obstructive pulmonary disease commonly observed in these individuals, is also discussed.

In this article, we also examine the association of various HIV proteins with pulmonary vascular injury to understand the pathophysiology involved. In addition, we have summarized the known molecular mechanisms involved in the pathophysiology of HIV-PH based on cell culture and various animal model studies.

### High prevalence of HIV-associated PAH

The prevalence of group I PH, PAH in people living with HIV (PLWH) is alarmingly high compared to the general population. Using echocardiography as the primary diagnostic tool, a Swiss study determined that the prevalence of PAH was 0.5% in a cohort of 1,200 HIV-infected patients during the pre-HAART^1^.

A study that analyzed echocardiographic data collected from a large group of individuals enrolled in the Veterans Aging Cohort Study produced findings that may be useful in the monitoring and screening of HIV-infected patients who are at risk of developing cardiopulmonary complications. The study demonstrated that HIV-infected subjects with high viral loads (> 500 copies/ml) and low CD4 cell counts (<200 cells/ *μ*l) were more likely to exhibit a pulmonary artery systolic pressure (PASP) exceeding the 40-mmHg threshold than uninfected subjects^2^.

More recently and with antiretroviral therapy emerging as the standard of care, a multicenter study conducted in France found the prevalence of PAH to be 0.46% in a population of 7,648 HIV-infected patients^3^. The diagnosis of PAH in patients with unexplained dyspnea was made by measuring tricuspid regurgitant jet velocity and mean arterial pulmonary pressure using transthoracic Doppler echocardiography and right heart catheterization, respectively, according to the WHO requirement for defining PAH^4,5^. In contrast, the prevalence of PAH in the general population was 0.0015% based on the findings of a French national registry published a few years before the above study. However, smaller cohort studies that relied exclusively on echocardiography to make a diagnosis of PAH have yielded even higher prevalence rates.

A cohort of 374 HIV-positive patients showed a prevalence of 6.1% PAH when determined by echocardiography^6^. Using a PASP of 40 mmHg as the diagnostic threshold, Isasti et al. found a prevalence rate of 2.6%^7^. Further, Quezada et al. defined a right ventricular pressure higher than 35 mmHg as PAH resulting in a prevalence of 9.9% in HIV patients^8^. In conclusion, PAH in PLWH is surprisingly high, even after the introduction of HAART.

## Risk factors that complicate HIV-PH

### Substance abuse

Illicit drugs are considered an important driver in spreading HIV infection worldwide. CDC data highlights that for every 10 new HIV infections, one infection is an individual who injects illicit drugs, accounting for 7% of new HIV infections in 2018^9^.

Although most cases in PAH remain idiopathic, exposure to drugs – including substance abuse – have been classified as one of the major risk factors for the development of Group 1 PAH in addition to HIV-1 infection. Certain drugs known to cause PAH are already withdrawn from the market e.g., aminorex, fenfluramine and its derivatives and benfluorex. Some stimulants and drugs of abuse like (meth)amphetamines, phentermine and mazindol are considered as “very likely” risk factors, whereas cocaine, phenylpropanolamine and interferon are considered as “possible causes” for PAH^10^.

The high probability of concurrent drug use in people living with HIV (PLWH) is correlated with a higher prevalence of HIV-associated PAH in individuals who are illicit drug users. There are several case-control studies, meta-analyses, and systematic reviews to support drug abuse or intravenous drug use (IVDU) as a risk factor for HIV infection of PAH^11,12^, reporting as high as 59%^13^, 62.5%^14^, 72%^15^, and 90%^16^.

A study by Himelman et al. reporting PAH in six HIV-infected individuals had three IVDUs^17^. According to a California-based prospective observational study, 42% were found to be IVDUs and 20% stimulant drug users (cocaine and methamphetamine) among the 35% of total HIV-PAH patients with greater than 30mmHg PASP^18^.

In another retrospective French study on HIV-PAH patients, 57% of HIV-PAH patients diagnosed with stage IV PAH were found to be IVDUs^19^. Later, a systemic review based on 180 case reports, concluded the overall prevalence of HIV-PAH in 0.5% HIV-infected patients of which 49% had IV drug use as the main risk factor^20^. In 2014, a single-center retrospective study from Spain on 18 HIV-PAH patients, concluded IVDU as a major risk factor in 77.8% of HIV-PAH patients presenting with NYHA functional class II-IV of PAH^21^.

Mondy et al. in the multi-center SUN Study on HIV-infected individuals on HAART reported a prevalence of 57% patients with mild/moderate to severe PAH, with right ventricular pressures greater than 30 mmHg. Among these, 30% were reported to be using cocaine, heroin, or methamphetamine, and an additional 30% were reported to be addicted to marijuana^22^.

A cross-sectional study from Tehran suggested HIV-PAH with PASP > 30 mmHg in 3% of total HIV-infected patients among which 50% were IVDU users^23^. Furthermore, Schwarze-Zander et al. reported a 6.1% prevalence of PAH in asymptomatic HIV-infected individuals; with history of IVDU significantly associated with a higher prevalence of HIV-PAH (*p* < 0.001)^6^. In general, IPAH is more prevalent among females, but among the drug users who are HIV-positive, PAH is more prevalent among men, and this could be due to a higher percentage of men abusing illicit drugs.

### COPD-related pulmonary vascular disease

Since the advent of HAART, non-AIDS defining comorbidities, including damage to the lung parenchyma, have become predominant among PLWH. Recently, HIV infection was recognized as an independent risk factor for emphysema, in addition to the well-established role of cigarette smoking^24^. COPD/emphysema is the most commonly diagnosed chronic lung disease in HIV-positive individuals^24,25^ and with the advancing age of the HIV-infected population, it is becoming increasingly prevalent^26^.

Despite antiretroviral therapy (ART) and smoking cessation, the lung function of tobacco users with HIV continues to decline^27–29^. Given this role of HIV in COPD, it is not surprising to find an increased incidence of COPD and pulmonary hypertension/right-sided heart failure (Group III PH) in PLWH^30^.

The prevalence of Group III PH depends on the severity of the disease, but also on the definition of PH and the method of diagnostic assessment. Spirometry data derived from the Global Initiative for Chronic Obstructive Lung Disease stage IV showed that up to 90% of patients at this stage had mPAP > 20 mmHg, with the majority having pressures ranging from 20 to 35 mmHg. About 1–5% of COPD patients had mPAP> 35–40 mmHg at rest^31^.

In addition, exercise-induced PAH in COPD may be due to comorbid left heart disease. There are a cluster of patients exhibiting a “pulmonary vascular COPD phenotype”, characterized by less severe airflow limitation, hypoxemia, reduced lung diffusing capacity (*D*_LCO_), hypocapnia, and exercise limitations due to cardiovascular dysfunction^32^.

It has previously been established that the presence of PH has a stronger association with mortality in COPD than forced expiratory volume in 1s (FEV_1_) or gas exchange variables^33^. In addition, an enlarged pulmonary artery diameter, as detected by computed tomography (CT) scan predicts hospitalization due to acute COPD exacerbation^34^.

Are Group III PH -associated vascular dysfunction and endothelial damage the chicken or the egg in disease generation and progression? Although smoking is still the prevailing cause of the decline in lung structure and function, previous experimental studies have provided supporting evidence for the hypothesis that PH and emphysema may have common vascular (endothelial) commonalities. For instance, Seimetz et al. have shown that PH precedes emphysema formation in a cigarette smoking model of small rodents and that this commonality was based on endothelial iNOS upregulation and eNOS downregulation, which led to the loss of vascular structures mediated by programmed cell death (apoptosis)^35^.

Indeed, inducing endothelial cell death was found to be sufficient to cause both PH and emphysema in rodent models^36,37^. Furthermore, EMAP II, an endothelial cell-specific proapoptotic protein, was demonstrated to be both necessary and sufficient to cause an emphysematous phenotype in small rodents exposed to cigarette smoke^38^. Interestingly, endothelial-specific transgenic expression of the HIV accessory protein Nef (functions of HIV-Nef are discussed in more detail in a later section) can also cause pulmonary emphysema which is associated with Nef-induced EMAP II upregulation^39^.

The close association of vascular damage with both emphysema and PH formation is underlined by models showing the loss of microvascular structures in the lung parenchyma preceding changes in pulmonary arteries and PH^40^, thus further highlighting the complex nature of Group III PH. In this context it is noteworthy that even under moderate exercise conditions, COPD patients may show a rapid rise in mPAP, indicating loss of lung vasculature, vascular distensibility and/or vessel recruitment capability^30^.

### Left heart disease-related PH

Pulmonary hypertension due to left heart disease (LHD) under the 1998 classification was referred to as *pulmonary venous hypertension* and classified under Group II PAH^41,42^. Based on underlying etiology, Group II PH patients were generally classified under two main categories i.e., those with end-stage heart failure, or those with mitral valve disease. However, recently this was extended to heart failure (HF) patients with preserved ejection fraction (HFpEF)^43^. In the past, mitral stenosis was considered to be the major cause of PAH-LHD, however with the advent of valve-replacement surgeries and decrease in rheumatic heart disease, the focus of PAH-LHD has moved towards heart failure due to ischemia-reperfusion injury. These complications result in elevated filling pressure in the left atria and subsequently lead to a retrograde increase in pulmonary venous, arterial, and capillary pressures. Patients with PH due to LHD (PAH-LHD) show an increase in pulmonary artery pressure (PAP) secondary to increased pulmonary capillary wedge pressure (PCWP). In patients with PH-LHD, PCWP is found to be elevated (> 15 mmHg) and therefore it is often termed as post-capillary PH, contrary to Group I PAH, which is generally referred to as pre-capillary PAH^44^.

Cardiomyopathy with decreased left ventricular ejection fraction (LVEF) – with or without symptoms of heart failure – is considered a primary long-term cardiac complication in HIV patients. In addition to cardiomyopathy, HIV is also known to be a key risk factor for acute myocardial infarction (AMI), further leading to heart failure^45,46^. Nonetheless, studies have shown HIV represents a key risk factor for heart failure independent of AMI^47^.

With the advent of ART, the number of cardiomyopathy patients with systolic dysfunction has reduced and the number of patients with diastolic dysfunction are on the rise^48,49^. A study by Hsue et al. based on echocardiographic measurements of 192 HIV-infected patients and 52 uninfected individuals, found a higher prevalence of diastolic dysfunction and increased left ventricular mass in HIV-infected individuals associated with low CD4 nadir^50^.

The SUN Study (Study to Understand the Natural History of HIV/AIDS in the Era of Effective Therapy) that reported PAH in 57% HIV-infected patients reported LV systolic dysfunction in 18% and diastolic dysfunction in 26% of HIV-infected subjects^22^. In an observational veteran aging cohort study, increased risk of heart failure with reduced ejection fraction (HFrEF) and heart failure with preserved ejection fraction (HFpEF) were observed in HIV-infected individuals on HAART when compared to values from uninfected individuals. Lower viral load was noted to be a risk factor only for HFrEF, which was observed to manifest at a younger age compared to uninfected individuals^51^. However, both HFpEF and HFrEF were associated with post-capillary PAH, increasing the morbidity and mortality risk in both groups of heart failure^52^. Primarily PAH due to left heart failure involves increased left atrium and ventricular filling pressure as a consequence of compromised left ventricular systolic or diastolic dysfunction, leading to elevated pulmonary arterial pressure, vasoconstriction, and arterial vascular remodeling.

The mechanisms involved in left ventricular failure/dysfunction are multifactorial and may or may not involve myocarditis. Direct infection to the myocardium, opportunistic infections, nutritional disorders, autoimmune disorder, and the effect of ART are some of the underlying principles in left ventricular failure associated with HIV.

HIV patients are susceptible to opportunistic infections including *Cytomegalovirus, Nocardia* and *Cryptococcus*^53^. Direct infection to the myocardium by these may lead to altered LV function secondary to infection-associated pericardial effusion^54^. Reports of HIV infecting the myocardium are rare, but systemic inflammation secondary to HIV-1 results in the release of cytokines such as TNF-*α*, iNOS and IL-*β*, resulting in inflammation-induced cardiomyopathy in HIV patients^55,56^. In addition, HIV-infected individuals with low CD4 levels have a higher degree of systemic inflammation and immune activation associated with the risk of heart failure^57^. Several studies also correlate the role of HAART in children and adults with left ventricular dysfunction, which has been reported to cause a decrease in LV mass perhaps due to mitochondrial dysfunction^58,59^. Importantly, substance abuse – especially methamphetamine, cocaine, and alcohol – are generally considered to aggravate the risk of HIV-induced HF^51^.

In conclusion, LHD is found to be common among HIV patients and according to the newer hemodynamic definition of PAH with the criteria of mPAP > 20 mmHg, it is likely to see a rise in the prevalence of PH among HIV-infected population. Implementing the strategies to identify the individuals at risk for heart failure and PH are needed to minimize the prevalence of cardiovascular diseases in PLWH.

### Co-infections

In spite of the highly effective combination of anti-retroviral drugs which checks the viral load and reduces mortality and morbidity in PLWH, opportunistic co-infections are still one of the major causes of death in these individuals in countries with emerging economies. Opportunistic infections are disproportionately higher in HIV-infected individuals from sub-Saharan Africa, including hepatitis B virus (HBV), hepatitis C virus (HCV), *Cryptococcus neoformans*, and *Pneumocystis jirovecii.* Hepatitis C virus is known to increase the risk of pulmonary hypertension in PLWH^60^. In a cross-sectional study conducted on 6,032 veteran participants, it was found that individuals with co-infection of HIV/HCV had higher PASP than uninfected individuals^61^. Parikh et al.^62^ linked the association of increased prevalence of PAH in HIV/ HCV co-infected individuals with up-regulation of miRNA-21(miR-21) in plasma.

Pneumocystis pneumonia (PCP) remains a common diagnosis in HIV-infected adults^63^. The initial exposure to *Pneumocystis jirovecii occurs in childhood and presents as a self-limited, upper respiratory infection that triggers an immunogenic response*^64^. However, immunocompromised patients can become re-infected and present with low-grade fever, dyspnea, non-productive cough, elevated lactate dehydrogenase (LDH) serum levels, and ground-glass opacities on computed tomography (HRCT). Low CD4 cell counts, suboptimal antiretroviral drug coverage and a large pool of population devoid of basic healthcare facilities has made those from developing economies more vulnerable to the PCP.

Studies have shown high seroprevalence of *P.jirovecii* in African children^65^ and adults^66,67^. The gold standard for the diagnosis of PCP involves specimen collection via bronchoscopy with bronchoalveolar lavage (BAL), and the microscopic detection of cysts using immunofluorescence staining. In recent years, less invasive diagnostic tests have been implemented, including PCR assays, (1 →3)-beta-D-glucan serology testing and ELISA^63,64^.

Prophylaxis with trimethoprim-sulfamethoxazole is recommended in patients with a CD4 count below 200 cells/µl, or previous history of AIDS-defining illnesses, such as oral candidiasis, as well as in patients with a CD4 percentage below 14%^68^. Of note, following the introduction of ART, bacterial pneumonia has replaced PCP as the most frequent HIV-associated pneumonia in the United States^67,69^. ART and trimethoprim-sulfamethoxazole have been reported to reduce the risk of bacterial pneumonia, whereas lower CD4+ T cell count and cigarette smoking correlated with increased risks^68^.

Among parasitic diseases, schistosomiasis presents an enormous burden in underdeveloped countries. Schistosome eggs get lodged in tissues and induce granuloma formation responsible for the pathology of the disease. Cross-sectional studies have shown that schistosomiasis may increase the risk of HIV infection^70,71^. Infection with *Schistosoma mansoni* is also known to potently induce PAH, and coinfection with HIV in the African continent – where Schistosoma is endemic in certain areas due to infested fresh water resources – is expected to further increase the incidence of PAH.

However, studies addressing increased PAH in this patient population are still under way^72^. Importantly, schistosomiasis can also impair the response to antiretroviral therapy among HIV-infected patients and treatment of schistosomiasis in co-infected patients reduced HIV viral replication and increase CD4+ T cell count^72^.

### Molecular insights into HIV-PAH

Prior research has shown that pulmonary vascular remodeling, resulting in the development of HIV-PAH, is chronic inflammation and a bystander effect of HIV-proteins released by infected or latently infected macrophages and T cells^72–76^. Despite the use of several detection methods, like immunohistochemistry, electron microscopy, polymerase chain reaction and DNA *in situ* hybridization, attempts to identify HIV infection in the lung vascular cells have been unsuccessful^77,78^. Development of PAH in a non-infectious HIV-transgenic (Tg) rat model expressing HIV-1 proteins without active infection further highlights the bystander effect of viral proteins on the pulmonary vasculature^79^. Hence, the focus of the scientific community has mainly been on analyzing the role of HIV viral proteins in the pathogenesis of HIV-PAH^80,81^.

### Role of chronic inflammation

Although with HAART the mortality rate of patients with HIV has decreased significantly, and non-AIDS defining conditions have become the main cause of death among the aging population infected with the virus, an altered immune activity and low-grade inflammation is still seen in patients living with successfully controlled HIV viral loads. Therefore, aging and chronic inflammation have to be considered as contributory factors of pulmonary vascular diseases, and in particular of pulmonary hypertension.

Indeed, immune dysregulation has recently been associated with the pathogenesis of pulmonary arterial hypertension (PAH) of various etiologies. Following a pulmonary vascular insult, whether infectious, mechanical, or hypoxic; macrophages and lymphocytes occupy the perivascular space, participating in the process of vascular remodeling^82^. Both innate and adaptive immune systems are activated in HIV-infected patients therefore a low level of sustained immuno-inflammatory condition persists for many years even if the patient has controlled viral load.

The frequency of activated T cells, inflammatory cytokines and monocytes are higher in HIV patients compared to healthy subjects. Even a minor increase in these inflammatory biomarkers results in a significant sustained increase in risk for non-infectious disease-related morbidity^83^. Spikes et al. reported perivascular inflammation near the obliterated pulmonary vascular lesions in simian immune-deficiency virus -infected macaques along with increased MCP-1 and IL-8 in plasma of these macaques^84^. Even in PLWH who have a normal CD4 count, increased production of pro-inflammatory cytokines such as IFN-*γ* can alter the function of immune cells.

Lately, extracellular vesicles have also been implicated in mediating the cross-talk between infected inflammatory cells and pulmonary vascular cells^85^. Extracellular vesicles (EVs) are small bi-layered membrane bodies released from all cell types and can serve as a vehicle for transferring proteins, coding and non-coding RNAs, lipids, and metabolites between cells. EVs released by HIV-infected monocyte-derived macrophages are reported to potentiate pulmonary vascular endothelial injury and smooth muscle proliferation^85^, leading to the development of cardiovascular dysfunction^86^. Higher numbers of TGF-*β*-linked extracellular vesicles were reported in the plasma of HIV-PAH patients compared to uninfected and HIV patients without PAH. Furthermore, Nef-positive EVs have been shown to promote premature senescence and eNOS downregulation through Rac-1-dependent mechanism resulting in coronary endothelial dysfunction^87^.

Chronic HIV infection precipitates immune senescence by stimulating the clonal expansion of senescent T-cells, with a distinctive lack of the co-stimulatory protein CD28, and overexpression of CD57. Similar to what is seen in older, uninfected individuals, these senescent cells exhibit shortened telomeres, arrested cell proliferation, and increased production of pro-inflammatory cytokines^88^. Besides its effects on co-stimulatory signals, chronic HIV infection also causes an increase in the expression of co-inhibitory molecules, such as programmed death 1 (PD-1) on CD8+ T cells.

PD-1 is a potent modulator of T-cell exhaustion, a phenomenon that commonly arises after chronic antigen exposure and leads to loss of effector function. The level of PD-1 upregulation correlates with markers used to clinically assess HIV disease severity, including viral load and CD4^89^. Disease progression is also linked to upregulation of programmed death-ligand 1 (PD-L1). Compared to uninfected individuals, HIV-positive patients have higher levels of PD-L1 on antigen-presenting cells (APCs)^89,90^.

In the case of CD4+ T cells, PD-1, cytotoxic T-lymphocyte-associated protein 4 (CTLA-4) and other inhibitory receptors are highly expressed on the cell surface, thus contributing to the impaired function of CD4+ T cells in response to HIV infection^89^. Under normal conditions, the CD28 co-stimulatory pathway promotes IL-2-dependent expansion of T cells through epigenetic modifications. However, in CD4+ T cells expressing the senescence marker CD57, IL-2 secretion is suppressed by hypermethylation at CpG site 1 of the IL-2 promoter^91^. Along with the reduced CD4/CD8 ratio, triggered by HIV infection, senescent T cells represent a decline in immune function and an increase in the likelihood of opportunistic infections.

### Role of HIV Nef protein

HIV Nef is a viral protein expressed early in viral infection, that is, myristoylated at the N-terminus, with a relative molecular mass of 25–32 kDa, localized to the cytoplasm and inner face of the cell membranes^92–95^. HIV Nef has no enzymatic activity and requires myristylation for most of its known functions, which include restriction of immune surveillance, intracellular protein sorting, and phosphorylation of proteins. It is in the lipid rafts that Nef interacts with several host cellular proteins as an adaptor molecule. Several functional domains of Nef have been shown to be important for these effects, including a proline-rich (PxxP)_3_ domain, thought to interact with SH3 domains in protein partners, and 6-sequence domains that interact with the endocytotic cellular machinery, including a dileucine moti^96–98^. These domains are important for Nef-mediated downregulation of CD4 and blocking major histocompatibility antigen I (MHC I) trafficking to the membrane, allowing the infected cell to evade immune surveillance.

**Figure 1.**
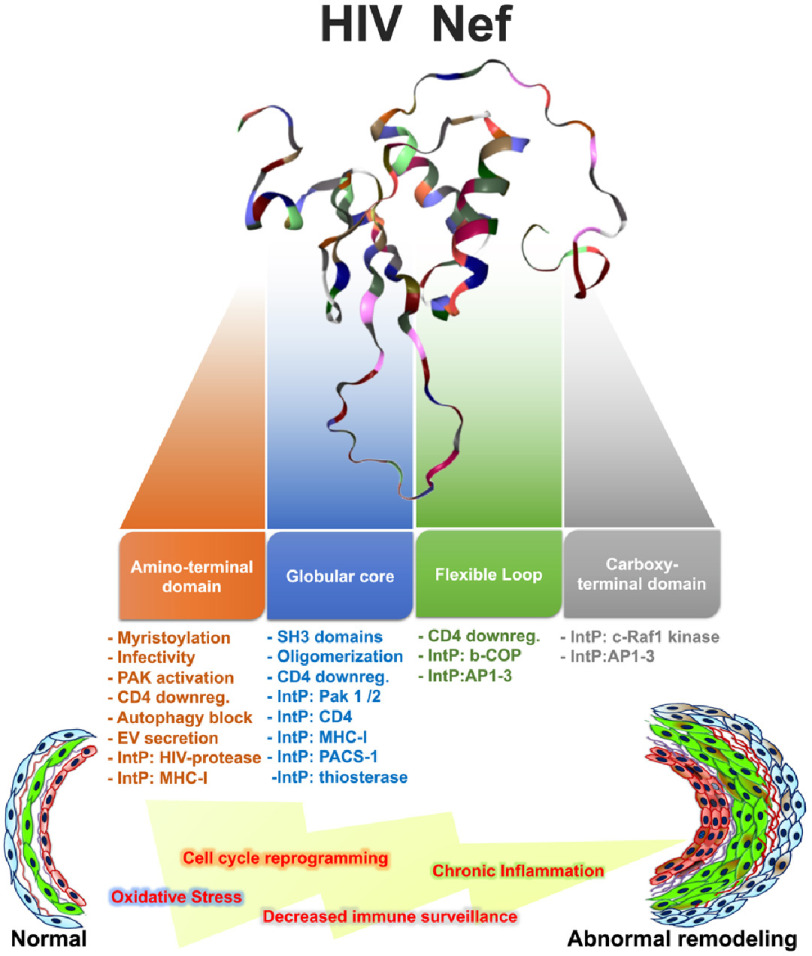
The multi-functionality of HIV Nef protein and its potential impact on the pulmonary vascular system.

Nef-mediated increases in macrophage inflammatory protein-1, IL-1 *β*, IL-6, and TNF-*α* in monocyte-derived macrophages (MDM) required the domains critical for the interaction with the endocytotic machinery because mutants (*i.e*., EE155-156QQ, and DD174-175AA) were ineffective^99^. Interestingly, Nef also induces de-differentiation of kidney podocytes^100^, possibly through cyclin-dependent mechanisms^101,102^, suggesting that Nef can influence cell proliferation and survival in a CD4-independent fashion. Importantly, mutations in the Nef PxxP motif diminish Nef signaling and the phenotypic changes in podocytes (Figure 1)^101,103^.

Nef is a vascular insult even in the absence of infection. It is well-established that endothelial cells (ECs) are resilient to HIV infection, however, the presence of cell-free Nef has been documented in vascular and perivascular cells. For instance, Nef induces apoptosis in brain endothelial cells when expressed intracellularly or exogenously^104^. Expression of HIV Nef increases ERK, MEK, and Elk MAP kinase activity, thereby affecting T cell activity, viral replication, and infectivity^105,106^.

The long-standing dogma is that severe PAH initiates with endothelial cell apoptosis followed by dysregulated endothelial cell proliferation. In the absence of infection, Nef enters lymphocytes via the human chemokine receptor CXCR4 and exerts apoptotic effects^107^. Nef also impairs vasomotor functions in pulmonary artery cells, decreases the expression of endothelial nitric oxide synthase and increases oxidative stress^108^. Moreover, HIV-infected lymphocytes or macrophages in the neighborhood of the pulmonary vasculature may release mediators, including viral proteins and cytokines that cause EC phenotypic alterations and uncontrolled proliferation. Nef can be detected found in cell culture supernatants (cell-free) when expressed in cultured cells or recombinant systems. *In vivo*, Nef circulates at 5–10 ng/mL plasma in HIV-infected patients^109^.

Alternatively, Nef-infected cells secrete Nef-containing exosomes that, in turn, induce cytopathic effects*.* The awareness regarding the transfer of viral components (infectious particles and/or proteins) between HIV+ cells and bystander uninfected cells, leading to pathogenic outcomes like oxidative stress, cancer, neurocognitive impairment, and immunological dysfunction in the uninfected cells is gaining momentum in the field^110–114^.

While the formation of conduits between T-cells may present a novel route for HIV transmission, the transfer of HIV proteins like Nef to B-cells is enough to impair adaptive immune processes such as antibody class switching^115^. Nef is one of the three immediate-early HIV genes which are still transcribed in HIV-infected cells, even in those receiving ART^116^.

If ART reduces HIV virion production, but not Nef gene expression, as previously described^116^, then the persistence of Nef expression might contribute to the higher risk of Nef-induced pathologies in patients receiving suppressive ART^117^. To this end, the Clauss group successfully found and reported significant levels of Nef-positive peripheral blood mononuclear cells (PBMCs) in ART-treated patients with HIV RNA viral loads < 50 copies/ml, compared to untreated controls^118^.

Because such a finding could be explained by the transfer of Nef from infected cells located in lymphatic tissues, further studies tested whether Nef from lymphatic or blood-derived mononuclear cells could also transfer to venous endothelial cells. Endothelial cells, especially in developing atherosclerotic plaques, are in direct contact with circulating HIV-infected cells and in a prime position for Nef transfer.

Using a co-culture approach with human umbilical cord vein endothelial cells (HUVEC) with PBMCs from viremic untreated HIV-infected patients for 24 h, the presence of Nef-positive endothelial cells with various levels of Nef positivity suggested that different levels of Nef transfer had occurred^118^. Together, those studies suggested that Nef protein may be widely transferred from HIV-infected cells to uninfected blood cells and bystander tissue cells, thus providing a means of pathogenic Nef activity, even when virus replication is controlled.

Sehgal and co-workers offered a sub-cellular explanation by establishing that Nef-induced disruption in protein trafficking pathways in endothelial cells and smooth muscle cells at the level of the trans-Golgi network is a pathogenetic mechanism in HIV-PAH^119^. Electron microscopy of plexiform pulmonary lesions characteristic of PAH, endothelial cells, fibroblasts, and smooth muscle cells in the lesions, showed enlarged endoplasmic reticulum, Golgi stacks, vacuolation and exocytic Weibel-Palade vesicles^120^, which suggested defects in intracellular trafficking.

Furthermore, they analyzed the structure of the Golgi apparatus in lung tissue sections of pulmonary vascular lesions in idiopathic PAH (IPAH) and in macaques infected with a chimeric simian immunodeficiency virus containing the human immunodeficiency virus (HIV)-*nef* gene (SHIV-*nef*) with histological evidence of proliferative, obliterative, and/or plexiform arterial lesions, using subcellular three-dimensional (3D) immunoimaging.

They noted aberrant Golgi fragmentation in cells containing HIV-nef-bearing endosomes and increased cytoplasmic distribution or dispersal of the Golgi tethers giantin and p115 in obliterative-plexiform lesions in patients with idiopathic PAH, and in macaques infected with SHIV”-nef^120^. Strikingly, the vascular cells that displayed increased dispersal in Golgi tethers were the Nef positive cells.

Another noteworthy finding was that the Golgi histopathology in the SHIV*nef* macaque lungs was indistinguishable to that found in idiopathic PAH. These studies from Sehgal and colleagues investigating the Golgi apparatus ignited discussions about potential relationships between disrupted endothelial cell membrane trafficking and mechanisms of PAH.

While PAH is highly prevalent in HIV patients, not every patient with HIV develops PAH. The characterization of viral, genetic, or environmental (lifestyle) that contributes to PAH is (and shall continue to be) the subject of intense investigation.

The Flores group in Colorado, USA hypothesized that certain sequence motifs would be more prevalent in alleles from HIV-infected patients with PAH than in HIV-infected normotensives. Patient cohorts from France (Drs. Marc Humbert, Gerald Simmoneau, and Cecile Goujard), Italy (Dr. Nicola Petrosillo), and California, USA (Drs. Priscilla Hsue and Laurence Huang) were investigated. Briefly, HIV *nef* gene was sequenced from frozen PBMC DNA, plasma and bronchoalveolar lavage fluid from these patients. Results showed that Nef polymorphisms found in human Nef were similar to those found in SHIV-nef-infected monkeys. Furthermore, bioinformatic analyses of the Nef alleles present in the French and California patient cohorts uncovered Nef polymorphisms in HIV-PAH that were statistically significantly different from the normotensive individuals in each group^121^.

Specifically, 10 Nef polymorphisms were reported in the French PAH group: the PxxP motif (proline-rich area essential for Nef interaction with SH3 domain-containing proteins^122^, where x is any amino acid), the L_58_V CD4 down-regulation domain^94^, the E_63_G acidic cluster mediating the sequestration of MHC-1 in the *trans-*Golgi network^123^, and the M_79_I/T_80_N/Y_81_F phosphorylation site for protein kinase C^124^.

Additionally, changes were reported near M_20_ – whose functional role is to interact with the adaptor protein 1 (AP-1) and efficiently prevent MHC-1 trafficking to the membrane^125^. Importantly, seven of these ten *nef* polymorphisms were validated in the San Francisco group with HIV-PAH^121^.

Using ≥2 polymorphisms in Nef functional domains as a cutoff, whether the length of HIV infection, age, or ART would have an impact on the selection of Nef variants was also investigated. There was no correlation between the number of polymorphisms and the length of HIV infection or age. In addition, ART is not associated with the presence of > 2 Nef variants in subjects with PAH from Europe or San Francisco. Although the Nef variants identified tended to cluster together: L_58_V-Y_81_F, PxxP-A_53_P, PxxP-H_40_ and A_53_P-H_40_Y in 4 subjects and Y_81_F-PxxP in 5 subjects, contingency analyses showed that there was no particular mutation associated with the presence of ART^121^.

### Role of HIV Tat protein

HIV-1 Tat (Transactivator of transcription) is a basic non-structural protein that recruits positive transcription elongation factor b (P-TEFb) of the host to a transactivation response element (TAR) in the RNA stem-loop, thereby enhancing the transcription of HIV-1. Tat is made up of five distinct domains: cysteine-rich, core, N-terminal, fundamental, and C-terminal. In addition to its intracellular function of enhancing transcription of the viral promoter, approximately two-thirds of the Tat is secreted by the infected cells and acts extracellularly by binding to a number of neighboring and distant cells. Tat export has been reported to be dependent on the binding of its basic domain with phosphatidylinositol-4,5-bisphosphate (PtdIns(4,5)P_2_) at the plasma membrane of T cells^126^.

Extracellular HIV-Tat can bind integrins and vascular endothelial growth factor receptors via the c-terminal domain, containing an Arg-Gly-Asp (RGD) sequence and/or a basic domain^127,128^. Tat, on binding to these receptors, triggers signaling pathways that affect diverse processes, culminating in the pathogenesis of several HIV-associated co-morbidities – ranging from pulmonary hypertension to cognitive abnormalities^129,130^. Previous studies suggest that Tat acts as a proto-cytokine, which modulates the key functions of various cell types, including endothelial cells (ECs) as well as smooth muscle cells (SMC)^76^. HIV-Tat displays a dual function with respect to cell survival and cell death based on the micro-environment^131^. Furthermore, Tat has been reported to induce EC senescence through up-regulation of miRNA 34a and miRNA 217 and inhibition of SIRT1 expression^132^.

**Figure 2.**
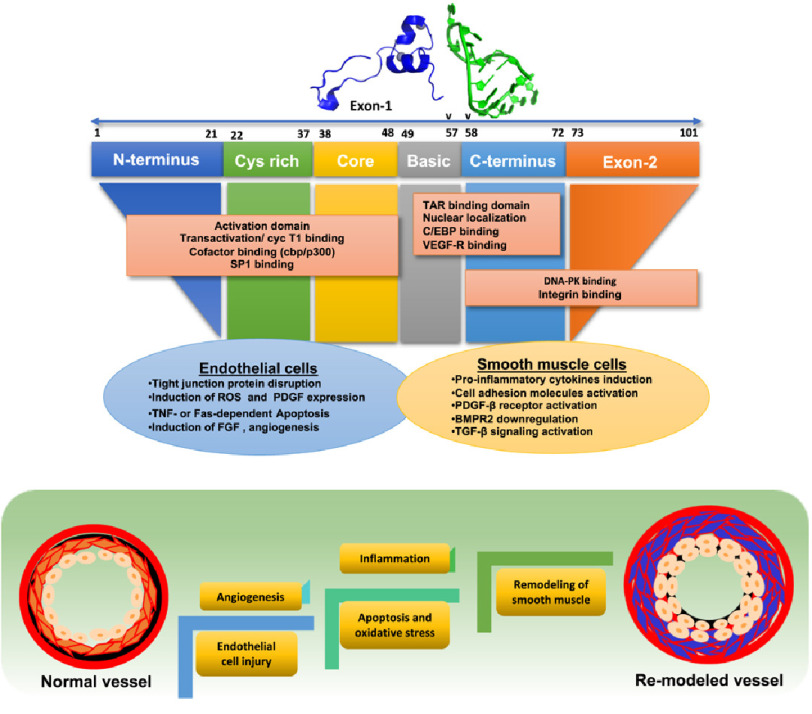
Depicts how different domains in Tat proteins affect pulmonary vascular remodeling via endothelial cell dysfunction and smooth muscle proliferation.

Being an angiogenic factor is a key feature of Tat protein, and by enhancing the endothelial differentiation and tumor angiogenesis, it plays a vital role in HIV-associated Kaposi sarcoma. Tat specifically attaches to VEGF-A tyrosine kinase receptor (Flk-1/kinase inert domain receptor (KDR)) and activates a downstream signaling cascade to enhance angiogenesis^128^ by inducing basic fibroblast growth factor and integrin pathways. HIV Tat, *via* its interactions with *α*_v_*β*_3_ integrin, stimulates focal adhesion kinase and NF-*κ*B activation that leads to endothelial cell proliferation^133–136^. Furthermore, activation of Ras/Rac1/ERK pathway is also implicated in the Tat-mediated proliferation and survival of endothelial cells^137^. This was later reported to be NADPH oxidase 4 (NOX4) dependent. However, Tat-mediated NOX2 dependent Rac1/JNK activation has been observed to regulate actin cytoskeletal dynamics of endothelial cells (Figure 2)^138^.

Tat-induced reactive oxygen species (ROS) are also known to promote HIF-1 *α* accumulation in pulmonary endothelial cells, leading to enhanced expression of a smooth muscle mitogen, platelet-derived growth factor (PDGF)^139^. In addition, Tat augments pro-oxidative conditions by reducing glutathione levels^140^ and weakening the expression of Mn-superoxide dismutase (Mn-SOD), a mitochondrial superoxide scavenger^141^.

Contrary to its role in promoting cell survival, Tat also is known to augment apoptosis of primary microvascular endothelial cells via triggering either TNF-*α* or Fas-dependent pathways^142^. Induction of apoptosis by Tat is not only mediated by activation of extrinsic pathways through apoptotic ligands, but also by activation of intrinsic pathways through direct entry of Tat^143^. This is supported by findings showing Tat-mediated phosphorylation of Erk 1/2, caspase-3 activation, and apoptosis of coronary artery endothelial cells^144^. In addition, numerous studies have implicated ROS-mediated activation of ERK1/2/MAPK signaling in altering the composition of tight junction proteins, thereby increasing endothelial permeability^145^. Our group reported ROS-dependent induction of both autophagy and apoptosis during early treatments of pulmonary ECs with Tat. However, chronic exposure to Tat in the presence of opioids prevented the accumulation of cytotoxic levels of ROS due to chronic activation of autophagy, thereby aiding cells to adapt to a chronic stress^146^.

HIV-Tat is also involved in the release of pro-inflammatory cytokines including MCP-1, IL-1 *β*^147^ and increases in the expression of cell adhesion molecules, such as E-selectin, ICAM-1 and VCAM-1 in ECs^148,149^. It is known to stimulate the ICAM-1 expression by suppressing miR-221/-222 via an NF-kB-dependent pathway^150^, while stimulation of VCAM-1 expression involves induction of p38 MAP kinase and NF-kB signaling^151^. Upregulation of these molecules results in the adhesion of monocytes and T-cells to the endothelium facilitating vascular injury^152–155^.

Multiple signaling pathways, such as activation of PDGF signaling, have been shown to enhance smooth muscle proliferation and progression to PAH^156^. Tat is reported to enhance the proliferation of pulmonary arterial smooth muscle cells via activation of PDGF-*β* receptors^157^. It further synergizes with cocaine in promoting smooth muscle hyperplasia through ligand-independent phosphorylation of PDGF *β* receptor at Y934 residue^157^. Our group demonstrated that Tat-mediated down-regulation in the expression of anti-proliferative bone morphogenetic protein receptor (BMPR) in pulmonary arterial smooth muscle cells further worsened in the presence of cocaine^158^.

This was reported to be mediated by miR-216a- and miR-301a-dependent translational repression^159^. Given that reduced expression or function of BMPR-2 signaling is known to exaggerate the proliferative TGF-*β* signaling, a parallel increase in the TGF *β*R1 and TGF *β*R2 expression and activation of SMAD-dependent and SMAD-independent downstream signaling cascades were observed in response to the combined treatment of cocaine and Tat.

### Role of HIV gp120 protein

The Envelope glycoprotein (Env) is a viral protein present on the surface of HIV virions. Extensive research efforts have investigated the use of HIV-Env as a tool to fingerprint the virus, as well as for the development of anti-HIV vaccines. The HIV envelope glycoprotein-120 (gp120) is essential for viral attachment and fusion through the host cellular membrane.

HIV enters the cells via interactions with CD4 receptors in the host cell and C-C chemokine receptor-5 (CCR5) and C-X-C chemokine receptor-4 (CXCR4). The CCR5 is a receptor for RANTES/CCL5, MIP-*α*/CCL3, and MIP-*β*/CCL4 in primary macrophages^160^. The CCR5 receptor is expressed in microglia, T lymphocytes, macrophages, and dendritic cells (DC). On the other hand, CXCR4 is a 7-transmembrane G protein-coupled receptor used by HIV as a co-receptor for preferential entry to T cell lines^161^.

Its natural ligand is stromal-derived factor-1 (SDF-1/CXCL12)^162^. Conventionally, HIV virions that use CCR5 as a portal of entry are designated as “R5”, while virions using CXCR4 are referred to as “X4”. The HIV preference for CCR5 co-receptor switches to a preference for CXCR4 over the course of HIV infection; this co-receptor switch predicts progression to AIDS in ∼50% of HIV+ individuals^163^.

The value of HIV gp120 as a molecular fingerprint has shed light on the lung as a potential reservoir for HIV. For instance, sequence analyses of the V3 loop of gp120 and pro-viral DNA copy numbers revealed that temporal evolution of gp120^164^ and that gp120 in bronchoalveolar lavage fluid and alveolar macrophages evolve separately from those in the peripheral blood^165,166^.

Early work from Kanmogne established that these cells do not express HIV coreceptors and discounted them as potential reservoirs for HIV^167^. However, accumulating evidence shows gp120-associated effects on vascular and pulmonary resident immune cells. HIV gp120 is a long-standing activator of lymphocytes^168^, and acts as viral superantigen by triggering the release of cytokines critical for T(H)2 polarization from human Fc*ϵ*RI+ cells^169^. HIV gp120 also facilities pathogen co-colonization in the host, as it inhibits fungistatic activity of bronchoalveolar macrophages against Cryptococcus neoformans^170^ and Pneumococci^171^. It also promotes Mycobacterium avium growth in alveolar macrophages by enhancing prostaglandin E2 release^172^, as well as Mycobacterium tuberculosis replication^173^.

Besides facilitating the viral attachment into the host cell, gp120 exerts several cellular effects. It induces the release of IL-1 *β* from macrophages in a time- and concentration-dependent manner;^174^ side effects also include apoptosis and increases the secretion of endothelin-1 from human monocytes^175^, astrocyte-brain microvascular endothelial cell co-cultures^176^, and primary lung endothelial cells^177^.

**Figure 3.**
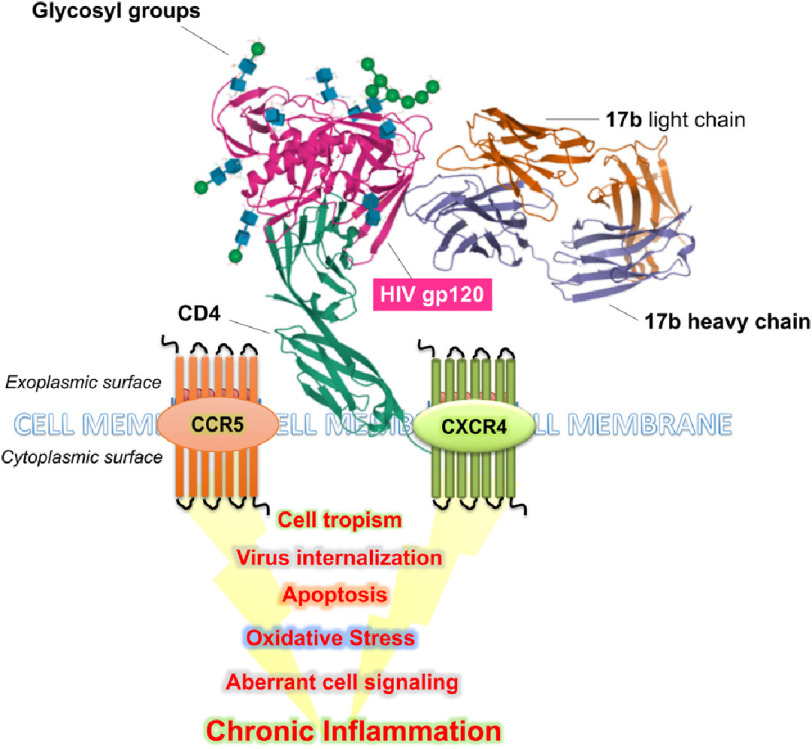
Depicts interaction of gp120 with CCR5 and CXCR4 and downstream molecular alterations.

Interestingly, the apoptosis induction of gp120 in endothelial cells is mediated by the upregulation of EMAP II and its receptor CXCR4 on the surface of lung microvascular endothelial cells (Figure 3)^178^. In addition, excessive mucus formation, which is a feature of chronic bronchitis, COPD, and asthma, is also induced and regulated by HIV-gp120 in the airway epithelial cells through the CXCR4- *α*7-nAChR-GABAAR pathway^179^. Furthermore, the use of cocaine also enhances gp120-induced pulmonary endothelial cell dysfunction^180^.

HIV gp120 effects are also complicated by aging. Studies using mouse primary lung fibroblasts exposed to HIV gp120 showed the induction of *α*-SMA expression and fibroblast-to-myofibroblast trans-differentiation by activating the CXCR4-ERK1/2 signaling pathways^181^, which may complicate fibrotic changes associated with aging.

## Understanding HIV-PAH Through the Lens of Animal Models

Animal models provide an enormous advantage in the screening of drugs and understanding disease mechanisms. The ideal pre-clinical PAH model should exhibit characteristics of human pulmonary hypertension, such as elevated right ventricular pressure and enhanced pulmonary vascular remodeling and right ventricular hypertrophy.

### Recapitulating HIV-PAH in non-human primates

In the wild, some non-human primate species are susceptible to infection with the simian homolog of HIV, the simian immunodeficiency virus (SIV). These primates, when infected with SIV, do not progress to simian AIDS, but instead host the virus as a non-pathogenic parasite. These “natural hosts” for SIV include sooty mangabeys (*Cercocebus atys*), African green monkeys (*Chlorocebus sabaeus*), mandrills (*Mandrillus sphinx*), sun-tailed monkeys (*Cercopithecus solatus*), and chimpanzees (*Pan troglodytes*).

The infection profile in the natural SIV hosts shows extremely high levels of SIV replication with no immunodeficiency (unchanged CD4+ T cell counts) and very low levels of cellular activation. The understanding on how the immune system of these primates resists being a target for HIV is the subject of intense research endeavors^182,183^.

In stark contrast (and closer to humans), there are macaque species that surrender to SIV infection. These “non-natural SIV hosts” include rhesus macaques (*Macaca mulatta)*, pig-tiled macaques (*Macaca nemestrina)*, cynomolgous monkeys (*Macaca fascicularis)*, and baboons (*Papio papio).* Rhesus macaques are the non-human primate of choice in human pathology research because of their major anatomical and physiological similarities to humans^184–187^.

Groundbreaking work from Chesney & Allen in 1973 reported that stumptail monkeys (*Macaca arctoides),* intoxicated with monocrotaline, presented *cor pulmonale*, with pulmonary vascular lesions that included fibrin and platelet thrombi within capillary lumina, hypertrophied arteriolar endothelium, as well as medial hypertrophy^188^. The unique ability to track disease development in non-human primates created the jumping board to further generations of animal models of PAH.

Rhesus macaques infected with SIV display systemic arteriopathy, suffered by at least 22% of infected macaques dying with simian AIDS. Chalifoux and colleagues described sclerotic lesions in large and medium-sized pulmonary arteries, with intimal thickening by smooth muscle cells and collagen^189^. The presence of these lesions was not associated with survival time, age, or sex; moreover, they were restricted to the pulmonary parenchyma in 37% of the animals. Systemic vasculopathy was evident by the presence of similar lesions in the kidney, heart, lymph node, intestinal tract, pancreas, and meninges, albeit less severe. Generalized arteriopathy was also documented in SIV-infected macaques in Yanai et al. studies^190^, in which lesions were characterized by intimal thickening and fibrosis with various degrees of vasculitis in macaques infected with SIV.

SIV and HIV sequences are not identical; therefore, the derivation of conclusions from SIV-based studies on human diseases must proceed with caution. The use of chimeric approaches has maximized the use of these animal subjects in HIV research, helping scientists to understand HIV gene evolution, evaluation of anti-HIV vaccines and the discovery of previously undescribed roles for HIV genes. Essentially, the chimeric approach consists of recombining viruses by substituting the gene of interest of an infectious SIV molecular clone with its equivalent in HIV. The resulting chimeric virions are designated SHIV^191^. The use of these animal models has been especially compelling to research in long-term complications of HIV infection, including pulmonary vascular remodeling and pulmonary hypertension.

One of the chimeric models described in the literature is the SHIV*nef* macaque model. The Desrosiers and Luciw groups documented that the S(HIV)nef macaque model is susceptible to AIDS^192,193^. This model was initially generated by infecting rhesus macaques with a molecular clone that resulted from the replacement of the SIV-*nef* gene (strain SIV _mac239,_GenBank # M33262) with the HIV-*nef* from a cloned virus isolated from an HIV-infected patient (SF-33, GenBank # AY352275) to create a recombinant SHIV*nef* (GenBank # AF490445)^192^. Intravenous inoculation of the macaques resulted in the animals dying of simian AIDS. Genetic characterization of the *nef* sequences in the animals showed Nef evolution, with at least four consistent amino acid changes acquired during passage **in vivo**^192^, of which two were later confirmed in a well-established cohort of individuals living with HIV and PAH^121^.

Retrospective histological analyses of the lung tissue in the same macaque cohort revealed the presence of luminal obliteration, intimal disruption, medial hypertrophy, thrombosis, and recanalized lumina, which are features beyond systemic arteriopathy^194,195^. Noteworthy, the cells within plexiform lesions were factor VIII+ and smooth muscle actin+. Moreover, monkeys infected with the parental SIV_mac239_ lacked pulmonary lesions. This finding was the first in the literature to suggest an association between HIV Nef and plexiform lesions in macaques. One consequence was the potential use of primates as models for HIV-associated pulmonary vascular pathology, as the plexiform lesion in monkeys were histologically indistinguishable from those in severe human PAH.

The central role of Nef was established with histological Nef colocalization to endothelial cells in macaques with severe remodeling and in patients with HIV-associated PAH^194,195^. The leverage of S(HIV)nef-infected macaque lung samples also allowed investigation of the mechanisms at the sub-cellular level. Studies from the Sehgal group uncovered dramatic cytoplasmic dispersal of giantin and p115, which are indicative of significant Golgi disruption^120^.

The same study corroborated findings in retrospective samples from patients with idiopathic PAH. Further studies by the same group showed that Golgi fragmentation in macaque endothelial and smooth muscle cells in pulmonary lesions was exclusive to cells containing Nef endosomes^119^.

Besides the formation of pulmonary vascular plexiform lesions and selective pressures on *nef,* SHIV-nef-infected primates also recapitulate features of idiopathic, familial, scleroderma-associated, and HIV-PAH like hematopathologies, changes in cardiac biomarkers indicative of ventricular hypertrophy, increased levels of interleukin-12 and GM-CSF, as well as decreased sCD40 L, CCL-2, and CXCL-1 in plasma^196^. Altogether, these studies establish the utility of SHIV-nef macaques as models of HIV-PAH.

Non-human primates have also been instrumental in researching the additive effects of drugs of abuse on HIV-mediated pulmonary vascular injury. For instance, rhesus macaques infected with neurovirulent SIV strain macR71/17E and treated with morphine for up to 59 weeks displayed severe pulmonary arteriopathy consistent with severe angioproliferative PAH in humans. The authors concluded that morphine potentiates the effects of HIV/SIV in advancing pulmonary arteriopathy^84^.

The Norris group performed echocardiography, and computed tomography (CT) scans, and right heart catheterizations in cynomolgus macaques infected with SHIV_89.6P_-*env* and rhesus macaques infected with SIV ΔB670. Their results showed that all the infected animals (with either SIV or SHIVenv) had increased right ventricular and pulmonary arterial pressures, with no evidence of systemic hemodynamic alterations^197^.

Longitudinal hemodynamic studies showed the development of either progressive or transient PAH, as well as increased pulmonary vascular collagen deposition in PAH animals^198^. In addition, there is a case report of two pigtailed macaques who died suddenly while chronically infected with R5-tropic SHIV strain. The authors reported total occlusion of the pulmonary artery by massive fibrin thrombus in both cases, as well as pulmonary vascular lesions similar to human PAH^199^.

A layer of complication is the use of viruses with different genetic backgrounds in different macaque species. While using animal models has significantly improved our understanding of the host-pathogen interactions in end-organ diseases beyond **in vitro** work, another limitation to the use of chimeric viruses is that it allows for the study of individual HIV genes/proteins in monkeys. Whether the response of the macaque as a host to a pre-selected virus, and vice-versa, is directly extrapolatable to human HIV isolates remains unaddressed. While their use in HIV-mediated pulmonary vascular diseases is appealing, macaque research is becoming difficult to justify. Nonetheless, non-human primates have revolutionized the field of PAH research because of their capability to allow measurements and interventions that are difficult or impossible to perform in humans.

### HIV-PAH in transgenic rats

Sugen-hypoxia and monocrotaline-induced inflammation-based rodent models are the most widely used models to understand the pathogenesis of PAH^200^. Attempts have been made to create an HIV-PAH model using non-infectious NL4-3 Δ gag/pol HIV-1 transgenic (Tg) rats. Deletion of gag and pol regions of the viral genome allows this model to express seven out of nine viral proteins i.e., env, tat, nef, rev, vif, vpr and vpu^201^. These HIV Tg rats exhibit most of the clinical manifestations observed in HIV-infected humans, including cardiac pathologies such as myocarditis and cardiomyopathy, and renal abnormalities such as proteinuria and nephrotic syndrome. Neurological changes, such as degeneration of peripheral nerves, cataracts and respiratory depression are also observed in these rats^201^.

Lund et al. demonstrated the development of pulmonary vascular remodeling and PAH in HIV-Tg rats^202^. Rats showed increased proliferation of pulmonary arterial SMC, increased PA medial thickness, RV hypertrophy and increased RVSP. Age plays a significant role in the heightened endpoints of PAH in the HIV-Tg rat model, as a significant difference was observed in PAH pathology between four and nine-month-old rats^202^. This study did not find plexiform lesions in HIV Tg rats and HIV proteins may prime the lung vascular endothelial cells for a second hit, which may lead to their proliferation.

The Sugen-Hypoxia model - and several other studies - suggest that a single assault, either in the form of inflammation, hypoxia, or genetic manipulation, is not sufficient to elicit all characteristics of clinical PAH in an animal model. Therefore, the quest to develop a severe PAH rat model shifted towards introducing multiple environmental or genetic hits. This was tried by Sutliff‘s group by using hypoxia (10% O_2_) for four weeks as the second hit in HIV-Tg rats and demonstrated increased PVR, elevated RVSP with a 65% increase in vascular muscularization in HIV-Tg rats compared to WT^79^. However, contrary to the findings by Lund et al. that were performed at Lovelace respiratory center in Albuquerque, Sutliff’s group did not find an increase in RVSP and mPAP in HIV-Tg rats in the absence of hypoxia. It could be that the location of the Lovelace center at a higher altitude resulted in the hypoxic challenge, thereby contributing to potentiating the disease in these rats.

In addition to hypoxia, drugs of abuse like morphine/opioids, cocaine and methamphetamine have been tested as a highly potential second-hit to viral proteins in triggering vascular injury and development of HIV-PAH^10,203^. Cocaine treatment of HIV-Tg rats was reported to augment pulmonary vascular remodeling and increase mPAP and RVSP, when compared with HIV-Tg rats or wild-type rats not treated with cocaine^204^.

Hyper proliferative SMCs isolated from HIV -Tg rats treated with cocaine had decreased expression of anti-proliferative BMPR-2^204^, with a corresponding increase in the levels of phosphorylated TGF *β*R1 and TGF *β*R2^205^. In context, with the undetectable viral load in PLWH, it is quite likely that circulating viral proteins and chronic inflammation – and not the active viral infection – makes the pulmonary vasculature susceptible to remodeling on the second hit of environmental stressors, as demonstrated in the model of this non-infectious HIV-transgenic rats.

### Pulmonary vascular disease in HIV-transgenic mice

In contrast to the robust Sugen-hypoxia rat model of irreversible PAH with a single dose of SU-5416 followed by hypoxia treatment, mice dosed three times with SU-5416 and exposed to 10% oxygen for three weeks, were able to exhibit only moderately elevated right ventricular systolic pressures and neo-intimal thickening of pulmonary vessels. In addition, a major disadvantage of the mouse model of Sugen-hypoxia is its reversibility, i.e., changing animal from hypoxic to normoxic condition reverses the PAH pathology^206^. Furthermore, developing an inflammation-based mouse model using monocrotaline is also one of the biggest challenges. Mouse strains lack CYP3A, a key enzyme required to metabolize the monocrotaline to its active moiety de-hydro-monocrotaline^207,208^, and therefore fail to generate elevated pulmonary pressures. Nevertheless, deletion or overexpression of some of the molecular proteins and targets playing a key role in the development of PAH has been tried in mice, to confirm and validate their role in PAH pathogenesis. Over expression of IL-6^209^, 5-Lipoxygenase^210^ and 5HTT transporter (5HTT)^211^ or knock out/down of BMPR-II^212^, adenosine receptor^213^, prostacyclin synthase^214^ and vasoactive intestinal peptide (VIP)^215^ demonstrated few characteristics of PAH, but failed to generate full-blown or advanced disease of PAH.

Likewise, HIV-transgenic (Tg-26) mice also expressing seven out of nine HIV proteins similar to HIV-Tg rats, failed to generate robust key pathological features of PAH^216^. Although these Tg-26 mice had normal PA pressure, similar to wild-type mice, they showed modest endothelial dysfunction and pulmonary vascular remodeling^217,218^. Therefore, even though mice provide an option of genetic manipulations, they are more resistant to PAH in comparison to rats and another hit of stressors like hypoxia or illicit drugs may potentiate the vascular injury translating to right ventricle failure.

### Humanized mice as a new frontier in HIV-PAH research

While non-human primates closely mimic infectious and pulmonary vascular disease, smaller animals, like rodents, advance the field by bringing tractability and cost-effectiveness to the bench of the researcher. Infection and its associated immunological storms are, however, key ingredients in the recipe for HIV-PAH. Moreover, HIV is a human-tropic virus that only infects human cells.

The humanization of the mouse immune system has significantly accelerated knowledge of HIV immunopathogenesis^219,220^. In essence, the hu-BLT mouse model is the best-described small animal model in the field of HIV pathogenesis. Humanized BLT mice^221^ reconstitute the human immune system after transplantation with CD34+ hematopoietic stem cells in the bone marrow and implantation of human fetal liver and thymus tissue under the kidney capsule. The reconstitution results in the production of systemically disseminated human B cells, monocyte/macrophages, dendritic cells, and T lymphocytes. Human innate and adaptive immune responses are detectable in the peripheral blood of BLT mice 4-8 weeks post-engraftment^222^. This mouse model supports productive HIV infection for up to 17 weeks, as shown by plasma viral load analyses and viral DNA in the spleen, bone marrow, lymph nodes, thymus, liver, and  lung after intravenous, rectal, or vaginal infection^223–228^. In addition, infected BLT mice exhibit HIV latency when treated with antiretroviral therapy^229^. It has also demonstrated that CXCR4-utilizing HIV-1 _LAI_ replicates at high levels and depletes CD4+ T cells in blood and tissues in humanized BLT mice^230^.

Several small animal models have been successfully implemented over the years for research in severe pulmonary diseases, like the monocrotaline and the Sugen/hypoxia models. Nevertheless, they are not susceptible to HIV infection, which limits mechanistic studies in HIV-associated PAH within the framework of the inflammation invoked by infection. While mice with humanized systems are now established models for HIV research, its validity for pulmonary vascular studies warrant exploration.

The combination of hypoxia and Sugen generated one of the most well-established models of PAH today^231,232^. The presence of HIV proteins alone tends to decrease VEGF expression in SIV-infected macaques with pulmonary arteriopathy^84^ –and experimental models of PAH may employ combinations of known pulmonary vascular insults like [HIV proteins + morphine]^84^ or [Sugen + morphine]^233^, all of which recreate pro-angiogenic signaling as hallmarks of PAH.

Recent studies combined HIV with either hypoxia (10% oxygen) or Sugen to test the suitability of humanized mice for HIV-PAH studies. The results indicate that neither hypoxia nor HIV alone induced severe PAH in the mice, and that the combination of HIV and hypoxia promoted PAH with a 25% survival rate, and that the combination of HIV and Sugen also promoted a PAH phenotype in the mice, with significantly higher survival (100%) (unpublished data).

Histopathological examination of lung tissues in humanized mice with HIV-PAH showed significant inflammation at interstitial, airways, veins, and artery levels, with foamy cells. Quantitative analyses of the pulmonary artery thickness (adjusted by diameter) showed significant muscularization of the arteries. The authors concluded that HIV-Sugen combinations lead to a better model suited for PAH and pulmonary vascular research due to its significantly higher survival and pulmonary hemodynamics (unpublished data).

Humanized mice have humanized hematopoietic cells, but still have murine vascular cells. These studies create a critical and timely foundation for mechanistic studies of PAH in small animals, which demonstrate the versatility and high translatability of this animal model. It is ideal for HIV research efforts with an emphasis on the heart and lungs. Additionally, humanized mice co-infected with HIV and *Mycobacterium tuberculosis* (Mtb) led to descriptions of HIV+ cells within Mtb lesions in the lung and that HIV co-infection increases the proliferation rate of Mtb because the animal succumbs to CD4 depletion^228^. Most recent generations of humanized mice now allow for humanization of the immune system together with the implantation of human lung tissue. Such models are suitable for research with human pathogens like Zika virus, cytomegalovirus, Middle East respiratory syndrome coronavirus (MERS), and respiratory syncytial virus^234^.

Molecular mechanisms underlying PAH pathogenesis are complex and have diverse etiologies; therefore, it is challenging to have a gold standard for the animal model which can mimic all the salient features of PAH observed in the clinical setup. More importantly, translating the data obtained from these models to clinical interventions should be used with caution and continuous efforts to develop a more robust model are needed.

## Closing Remarks

Longer survival of HIV patients due to modern HAART has refocused biomedical attention to combat the increase in secondary, non-infectious, complications including pulmonary hypertension.

Complicating matters further, people living with HIV often have a history of drug abuse and exposure to co-infections; both increasing the capacity of HIV to induce pulmonary vascular injury leading to diseases such as PAH.

In an attempt to identify targets for the intervention of HIV-induced comorbidities, several key molecular mechanisms of HIV-induced pulmonary vascular disease have been identified. These involve HIV proteins; Tat, Nef and gp120, which by deregulation of endothelial survival and pro-inflammatory activities play a significant role in HIV-induced PAH.

Although non-human primate and small rodent models have been examined to understand the mechanisms and pathobiology related to HIV-PAH, each model has its own limitations and do not completely recapitulate the clinical features of the disease, justifying the need to develop new animal models. Having both molecular mechanism and complementary pre-clinical models in our hands, it should not take long to develop drugs targeting HIV-induced comorbidities, including PAH.

## Abbreviations

HIV: Human Immunodeficiency Virus
HAART: Highly Active Anti-retroviral Therapy
PLWH: People Living With HIV
PAH: Pulmonary Arterial Hypertension
PASP: Pulmonary Artery Systolic Pressure
COPD: Chronic Obstructive Pulmonary Disease
iNOS: inducible Nitric Oxide Synthase
eNOS: endothelial Nitric Oxide Synthase
EMAP II: Endothelial Monocyte Activating Polypeptide II
TNF-*α*: Tumor Necrosis Factor-Alpha
IL-*β*: Interleukin Beta
HRCT: High-Resolution Computer Tomography
PBMC: Peripheral Blood Mononuclear Cells
MHC: Major Histocompatibility Complex
*α*-SMA: alpha Smooth Muscle Actin
BMPR-2: Bone Morphogenic Protein Receptor-2
EC: Endothelial Cell
SMC: Smooth Muscle Cells
